# Ethnic Disparities in the Development of Sight-Threatening Diabetic Retinopathy in a UK Multi-Ethnic Population with Diabetes: An Observational Cohort Study

**DOI:** 10.3390/jpm11080740

**Published:** 2021-07-28

**Authors:** Manjula D. Nugawela, Sarega Gurudas, A Toby Prevost, Rohini Mathur, John Robson, Wasim Hanif, Azeem Majeed, Sobha Sivaprasad

**Affiliations:** 1 UCL Institute of Ophthalmology, 11-43 Bath Street, London EC1V 9EL, UK; manjula.nugawela@ucl.ac.uk (M.D.N.); sarega.gurudas.17@ucl.ac.uk (S.G.); 2 Department of Population Health Sciences, King’s College London, London WC2R 2LS, UK; toby.1.prevost@kcl.ac.uk; 3 London School of Hygiene & Tropical Medicine, Keppel Street, London WC1E 7HT, UK; rohini.mathur@lshtm.ac.uk; 4 Institute of Population Health Sciences, Queen Mary University of London, London E1 4NS, UK; j.robson@qmul.ac.uk; 5 Birmingham City School of Nursing and Midwifery, Westbourne Road, Birmingham B15 3TN, UK; hanif.wasim@uhb.nhs.uk; 6 School of Public Health, Imperial College London, London SW7 2AZ, UK; a.majeed@imperial.ac.uk; 7 Moorfields Eye Hospital NHS Foundation Trust, 162, City Road, London EC1V 2PD, UK

**Keywords:** type 2 diabetes, retinopathy, ethnicity, general practice, risk factors

## Abstract

There is little data on ethnic differences in incidence of DR and sight threatening DR (STDR) in the United Kingdom. We aimed to determine ethnic differences in the development of DR and STDR and to identify risk factors of DR and STDR in people with incident or prevalent type II diabetes (T2DM). We used electronic primary care medical records of people registered with 134 general practices in East London during the period from January 2007–January 2017. There were 58,216 people with T2DM eligible to be included in the study. Among people with newly diagnosed T2DM, Indian, Pakistani and African ethnic groups showed an increased risk of DR with Africans having highest risk of STDR compared to White ethnic groups (HR: 1.36 95% CI 1.02–1.83). Among those with prevalent T2DM, Indian, Pakistani, Bangladeshi and Caribbean ethnic groups showed increased risk of DR and STDR with Indian having the highest risk of any DR (HR: 1.24 95% CI 1.16–1.32) and STDR (HR: 1.38 95% CI 1.17–1.63) compared with Whites after adjusting for all covariates considered. It is important to optimise prevention, screening and treatment options in these ethnic minority groups to avoid health inequalities in diabetes eye care.

## 1. Introduction

Diabetic retinopathy (DR) is a major microvascular complication of diabetes [1,2]. DR can progress from no DR to sight threatening diabetic retinopathy (STDR) without any symptoms [3]. In the United Kingdom (UK), the prevalence of DR and STDR among people with type 2 diabetes (T2DM) is approximately 30% and 1.5%, respectively [4,5]. In absolute numbers, this equates to around 1.5 million people with DR and 140,000 with STDR. In the UK, the two largest minority ethnic groups are South Asian (7.5%) and Afro-Caribbean (3.3%) compared to the White ethnic groups who make up 86% of the population [6]. These ethnic minority groups are likely to develop diabetes from a younger age and have a higher prevalence of diabetes compared to the White counterparts [7,8,9]. Similarly, the prevalence of DR is higher in South Asian and Black ethnic groups in the UK and these findings are consistent with global literature [4,10,11,12,13,14,15,16]. These ethnic differences in prevalence of DR are often explained by earlier age of onset of diabetes, and suboptimal control of risk factors. However, there is little data on differences in the incidence of DR and STDR in these ethnic groups when compared to their White counterparts. Studies on incidence of DR and STDR are mainly reported from Clinical Practice Research Datalink (CPRD) where ethnicity records are missing in about a third of the people with diabetes or from regions that do not have sufficient ethnic minorities to study the incidence at a population level [4]. It is also unclear if any ethnic differences in incident DR and STDR are due to variations in control of modifiable systemic risk factors. 

Duration of diabetes is one of the strongest risk factors of DR [17,18]. In the UK, all people with newly diagnosed diabetes are referred for annual DR screening within 3 months of the screening programme being notified of the diagnosis [19]. People with diabetes registered in the same practice have equal access to the same group of general practitioners and access to care is free in the National Health Service (NHS). The population in East London consists of a high proportion of people from ethnic minority groups. Therefore, it is a suitable population in which to evaluate whether ethnicity is an independent risk factor for incidence of DR and STDR. 

The aim of this study was to determine ethnic differences in the development of DR and STDR, and to identify risk factors for DR/STDR development in people with newly diagnosed and prevalent T2DM at baseline. We focussed on the two main ethnic minority groups, South Asian and Black and then further analysed the sub-groups of Indian, Bangladeshi, Pakistani and Caribbean and African ethnic groups to identify the role of ethnicity as an independent risk factor for DR/STDR.

## 2. Materials and Methods

The Moorfields Research Management Committee approved the use of this fully anonymised UK dataset for model development and validation (SIVS1047) and further ethics approval was not required. Approval was also obtained from the Caldicott guardian of this anonymised dataset in Queen Mary University London (QMUL). Individual level patient consent was not obtained, as this was an observational study using de-identified data.

This cohort study was conducted using de-identified data from general practice electronic health records collected in three Clinical Commissioning Groups (CCGs) in East London, which include Newham, Tower Hamlets and City and Hackney. These data covered more than 98% of the GP-registered population in these CCGs. The data included demographic information, diagnoses, prescriptions, referrals, laboratory test results and clinical values. Diagnoses, symptoms and clinical values in this dataset were recorded using the Read code classification. 

We included all adults with a diagnostic Read code for T2DM during the period 2007–2017 and aged 18+ at study entry. Study baseline was defined as the date of the initial T2DM diagnostic read code for each person within the study start and end dates. Start date was defined as the latest of study start date (January 2007), 12 months after the patient’s current registration date, or the date the patient turned 18. Follow-up time end date is defined as the earliest date of transferring out of the practice, or latest data collection date or date of death or January 2017. People who were lost to follow up were censored at the date they left the study. People with Type 1 diabetes, gestational diabetes, other forms of diabetes were excluded.

People were classified as having newly diagnosed (incident) T2DM if their T2DM onset date was the same as their initial T2DM diagnosis date within the study start and end dates. If they had an earlier T2DM onset date than the initial T2DM diagnosis date within the study period, they were classified as people with prevalent T2DM. Self-reported ethnicity was identified using the relevant Office for National Statistic ethnic group classification in the electronic health record of each patient. Ethnicity was considered in two ways. First in three categories as White, South Asian and Black and then we further considered the subgroups of Indian, Pakistani, Bangladeshi, African and Caribbean. Chinese, other Asian, other Black and any other ethnic group were grouped as ‘Other’. A small proportion of people with missing or unknown ethnicity (1.8%) were not included in the analysis. Socio-economic deprivation was categorised into quintiles of the Townsend score, which is made up of unemployment, overcrowding and car ownership variables with the most affluent in quintile 1 and most deprived in quintile 5.

The first record of any DR and any STDR were considered as the outcomes. Read codes for classification levels of DR severity levels included both the American Academy of Ophthalmology International Classification and UK National Screening Committee classification [3]. Those who had severe Non-Proliferative DR, Proliferative DR, grading classification for proliferative DR-R3 or grading classification for maculopathy-M1, were identified as people with STDR. A person’s DR grade was defined as the DR severity level in the worst eye. 

Covariates that are commonly found to be associated with DR and STDR according to existing literature were considered in this study [17,18], and these included age, gender, hyperglycaemia, systolic blood pressure (SBP), duration of diabetes, body mass index (BMI), total cholesterol, high density lipoprotein (HDL), eGFR, antidiabetic medication, history of cardiovascular disease (ischaemic heart disease, heart failure, stroke, peripheral vascular disease, cardiovascular death, Acute Myocardial Infarction, Bypass graft/Angioplasty, Angina Pectoris, Cardiac Arrhythmia, Major ECG abnormality, Silent Myocardial Infarction, Congestive Heart Failure, Transient Ischaemic Attack, Arterial Event requiring surgery), statin prescription and antihypertensive medication prescription. These covariates were measured at baseline for each individual and the closest record to the index date (±6 months) was selected for clinical variables. 

Sperate analysis were undertaken for the two groups of study population, people with newly diagnosed and prevalent diabetes, and then for the two outcomes DR and STDR. Univariable statistics were used to describe the baseline characteristics of the study population overall and by ethnic group. Kaplan-Meier Survival estimates with log-rank test were used to observe any differences in DR and STDR development over time by different ethnic groups. Multivariable Cox regression was then used to identify any association between the ethnic groups and development of any DR or STDR. Cox regression models were developed separately among people with newly diagnosed and prevalent diabetes for the two outcomes of DR and STDR resulting four models. All four models were adjusted for all covariates considered in the study, and the effects of each covariate was reported with hazard ratios (HR) with 95% confidence intervals (CI). Risk factors for development of DR were identified using the statistical significance of each covariate in each fully adjusted model (*p* < 0.05). We further analysed the ethnic differences in developing DR/STDR by stratifying the exposure variable into the more detailed ethnic categories of Indian, Pakistani, Bangladeshi and African and Caribbean. All statistical analyses were performed using Stata V.16. 

## 3. Results

### 3.1. Overall Study Population 

There were 71,406 people with a T2DM record within the study period who were registered with a GP practice for at least one year period. Of these, we excluded those who had background retinopathy (*n* = 6926) and sight threatening retinopathy (*n* = 409) before the baseline. People who had history of anti-diabetic treatment before their T2DM onset date (*n* = 3318) and people who were prescribed two antidiabetic drugs or insulin on the T2DM diagnosis date (*n* = 1466) were excluded and these were defined as coding errors. Of the remaining 59,287 people, ethnicity was recorded on 98% people and there were only 1071 (2%) who did not have their ethnicity recorded (unknown or missing) and these were also excluded, leaving 58,216 people eligible to be included in the study of whom 32,652 were people with newly diagnosed T2DM and 25,564 people with prevalent T2DM at the study baseline with mean duration of diabetes 7.4 (SD 6.1) years. 

### 3.2. Baseline Characteristics of the Study Population 

The following results are presented separately for groups of study population with newly diagnosed type 2 diabetes (Table 1) and prevalent T2DM at baseline (Table 2). Among people with newly diagnosed T2DM, we observed incidence rates of 82.8 and 4.2 events per 1000 person years for DR and STDR, respectively. Among people with known T2DM, the incidence rates for DR and STDR were 135.4 and 16.0 events per 1000 person years, respectively (Table 3).

Table 1 shows the characteristics of 32,652 people with newly diagnosed diabetes at study baseline by different ethnic group. Townsend score was recorded in 99.7%, BMI was recorded in 91% and HbA1c level was available in 85.3%, SBP was recorded in 97%, total Cholesterol level was recorded in 94% and eGFR level was recorded in 93%. A greater proportion of South Asians 6536 (43%) were in the <45 years age group compared with White (16%), Black (25%) and other (25%) indicating early onset of diabetes among South Asians. Higher proportion of South Asians were in the most affluent group 4043 (26.4%) compared with other ethnic groups with 15–21% people in this group as shown in Table 1. Almost 70% of people in all ethnic groups had a BMI value that was greater than 25 kg/m^2^. Almost 50% of South Asians had lower blood pressure <130 mmHg compared with other ethnic groups with a proportion of people from 34–43% in the same blood pressure group. White ethnic groups had more than twice as high prevalence of CVD 1388 (16%) compared to other ethnic groups with prevalence of CVD ranging from 6–8%. 

Table 2 shows the baseline characteristics of 25,564 people with prevalent T2DM at the study baseline. In this group, Townsend score was available in 99.7%, BMI was recorded in 89%, HbA1c level was recorded in 89%, SBP was available in 98%, total Cholesterol level was recorded in 91% and eGFR level was recorded in 91%. A greater proportion of South Asians 3055 (25%) were in the youngest age group whereas a greater proportion of White ethnic groups with T2DM were in the oldest age group at baseline 1396 (20%). Duration of diabetes were similar across the ethnic groups with approximately 16% of people in 0 to <2 years duration, 27% in the 2 to <5 years group, 31% in the 5 to <10 years group, 26% in the >10 years group. 

### 3.3. Kaplan Meier Plots

**Figure 1:**
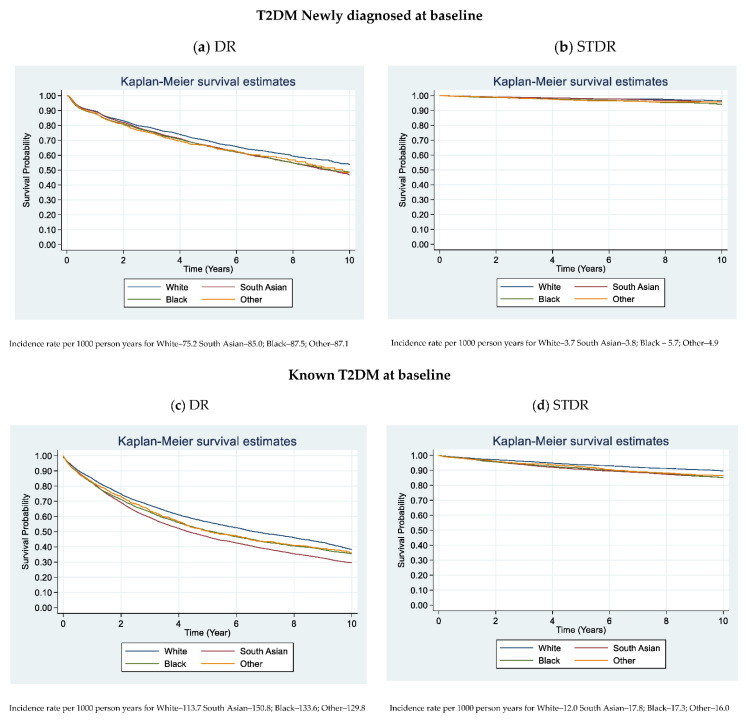
Kaplan-Meier Survival Estimates for DR and STDR.

Figure 1 shows the Kaplan-Meier Survival estimates for DR and STDR by different ethnic groups for newly diagnosed and people with prevalent T2DM at baseline. For DR, there was clear separation in survival probabilities over time by each ethnic group with the White group having a higher survival probability overtime compared to all other ethnic groups (log rank test; *p* < 0.001). This separation was even more visible in people with prevalent T2DM at baseline, White ethnic groups having the highest and South Asians having the lowest survival probability overtime (log rank test; *p* < 0.001).

For STDR, in the T2DM newly diagnosed group there was slight separation of survival probabilities overtime by different ethnic group (log rank test; *p* < 0.001), but there was a clear separation of survival probabilities among people with prevalent T2DM (log rank test; *p* < 0.001) with White ethnic groups having the highest survival compared with all other ethnic groups.

### 3.4. DR/STDR Events and Incidence Rate

Table 3 shows the number of incident cases of DR and STDR during the follow-up period and the incidence rate in each cohort. Among people with newly diagnosed T2DM at baseline, a total of 8638 people had DR events and 557 people had STDR events. For DR, White ethnic groups had the lowest incidence rate (75.2 per 1000 person years) and Black ethnic groups had the highest incidence rate (87.5 per 1000 person years). For STDR, White groups had the lowest incidence rate (3.68 per 1000 person years) and Black ethnic groups had the highest incidence rate of 5.66 per 1000 person years.

Among people with prevalent T2DM at baseline, a total of 12,124 had DR events and 2200 people had STDR events. For DR, White ethnic groups reported the lowest incidence rate (113.7 per 1000 person years) and South Asians reported the highest incidence rate of 150.8 per 1000 person years. In relation to STDR, White ethnic groups had the lowest incidence rate of 12.0 per 1000 person years and South Asian had the highest incidence rate of 17.8 per 1000 person years.

### 3.5. Multivariable Analysis and Ethnic Variation in Incident DR/STDR

Table 4 shows results for the multivariable Cox regression analysis for DR and STDR outcomes. Ethnicity was significantly associated with DR and STDR in both groups of people with newly diagnosed T2DM and prevalent T2DM at baseline after adjustment for all the covariates considered, which include age, gender, deprivation, BMI, Hba1c, SBP, Total Cholesterol, eGFR, history of cardiovascular disease, history of antidiabetic, antihypertensive and statin medication use.

**Figure 2:**
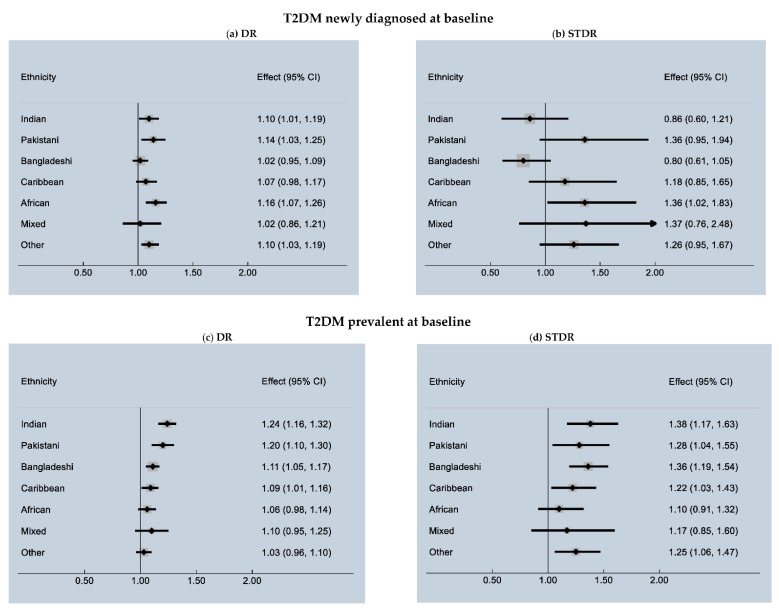
Risk of DR and STDR in ethnic minorities compared to white population.

Among people with newly diagnosed T2DM, Indian, Pakistani, African and ‘Other’ ethnic groups had significantly higher risk of DR compared to White ethnic groups with African having the highest risk (HR: 1.16 95% CI 1.07–1.26) (Figure 2a). However, in relation to STDR, only Africans (HR: 1.36; 95% CI 1.02–1.83) showed an increased risk compared to White ethnic groups (Figure 2b).

Among those with prevalent T2DM at baseline, Indian, Pakistani, Bangladeshi and Caribbean ethnic groups showed an increased risk of DR compared to White ethnic groups with Indian having the highest risk (HR: 1.24 95% CI 1.16–1.32) (Figure 2c). In the same group of people, Indian, Pakistani, Bangladeshi, Caribbean and ‘other’ ethnic groups showed a significantly higher risk of STDR compared to White ethnic groups with Indians having the highest risk of STDR (HR: 1.38 95% CI 1.17–1.63).

### 3.6. Other Risk Factors Contributing to DR and STDR

There were several other risk factors that were significantly associated with DR and STDR after adjustment for all the covariates considered in this study as given Table 4 and Table 5.

Among people with newly diagnosed T2DM at baseline, those who were in age <55 had significantly higher risk of DR compared to those who were aged 75+ (Table 4). Men were more likely to have DR and STDR compared to women with 12% (HR: 1.12 95% CI 1.07–1.17) and 22% (HR: 1.22 95% CI 1.02–1.47) increased risk, respectively. Those with HbA1c ≥ 100mmol/mol had highest risk of DR (HR:1.70 95% CI 1.57–1.85) and STDR (HR: 3.68 95% CI 2.73–4.95) compared to those with HbA1c < 50 mmol/mol. People with SBP ≥ 140mmHg had higher risk of DR (HR: 1.25 95% CI 1.17–1.33) and STDR (HR: 1.88 95% CI 1.45–2.44) compared to those with SBP < 120 mmHg. Total cholesterol, eGFR, CVD history and history of one antidiabetic drug use were not significantly associated with the risk of DR or STDR.

Among people with prevalent T2DM at baseline, those who had duration of ≥10 years diabetes had the highest risk of any DR (HR: 1.83 95% CI 1.71–1.96) and STDR (HR: 2.46 95% CI 2.06–2.90) compared to those with 0 to 2 years of duration (Table 5). People aged 45–54 years had the highest risk of DR and STDR compared to those who are aged 75+. Men had 10% (HR: 1.10 95% CI 1.06–1.14) and 15% (HR: 1.15 95% CI 1.06–1.26) increased risk of DR and STDR compared to women, respectively. Those with HbA1c ≥ 50 mmol/mol, SBP ≥ 120 mmHg, eGFR < 60 mL/min/1.73m^2^ had higher risk of DR and STDR compared to those in HbA1c < 50 mmol/mol, SBP < 120 mmHg and eGFR ≥ 60 mL/min/1.73m^2^, respectively. BMI, total cholesterol, CVD history and history of antihypertensive drugs were not significantly associated with the risk of DR or STDR.

## 4. Discussion

We investigated the ethnic differences in developing DR and STDR among people with newly diagnosed and prevalent T2DM using a primary care dataset from East London in which more than 98% of people with T2DM had complete data on ethnicity. From this dataset, over 58,000 people were eligible to be included in this study and among people with newly diagnosed T2DM, we observed incidence rates of 82.8 and 4.2 events per 1000 person years for DR and STDR, respectively. Among people with prevalent T2DM, the incidence rates for DR and STDR were 135.4 and 16.0 events per 1000 person years, respectively. These rates varied between different ethnic groups with higher incidence rates among South Asians and Africans compared to White ethnic groups. South Asian and Black ethnic groups had increased risk of any DR and STDR compared to White ethnic groups even after adjustment for age, gender, deprivation, BMI, Hba1c, SBP, total Cholesterol, eGFR, history of cardiovascular disease, history of antidiabetic drugs, antihypertensive and statins. Among people with newly diagnosed T2DM, Africans were 36% more likely to have STDR compared to White ethnic groups (HR: 1.36 95% CI 1.02–1.83). Among people with prevalent T2DM, Indians had the highest risk of any DR with 24% increased risk (HR: 1.24 95% CI 1.16–1.32) and STDR with 38% (HR: 1.38 95% CI 1.17–1.63) increased risk compared with White ethnic groups after adjusting for all the covariates considered. Overall, people with prevalent T2DM group with a mean duration 7.4 years at baseline showed stronger association between ethnicity and DR/STDR compared to the group of people with newly diagnosed T2DM diabetes at baseline.

In existing literature, there is only a small number of studies on incidence of DR and STDR, and most of them do not consider ethnicity as a possible risk factor. These studies included a UK study conducted among 1919 people with newly diagnosed diabetes and reported that 22% of the people developed retinopathy by the end of their follow-up period of 6 years [20]. The Liverpool Diabetes Eye Study was conducted among 4770 newly diagnosed T2DM people and 3.9% people developed STDR by the end of their 5 year follow-up period [21]. In another study with 16,444 people with T2DM, 16.4% developed DR and 2.7% people developed STDR after 10 years of follow-up [22]. Two recent studies by McKay eta al [23,24] also reported DR and STDR incidence among people with T2DM using the CPRD dataset. The first study reported 8263 (13.8%) of DR cases and 832 (1.4%) STDR cases during the 3.5 year and 3.8 year follow up periods, respectively. This was equivalent to an incidence rate of 39.2 cases per 1000 person years for DR and 3.6 cases per 1000 person years for STDR [24]. The second study assessed incidence of STDR among those with mild non-proliferative DR and a total of 1037 (5.5%) people developed STDR over a mean follow-up period of 3.6 (SD 2.0) years [23]. Although it is challenging to compare these studies, the event rates of both DR and STDR were higher in our study among both groups of people with newly diagnosed as well as prevalent diabetes at baseline. This is likely to be due to the higher proportion of minority ethnic group in our study population who have a higher risk of diabetes in general [7,8,9] as well as higher risk of DR as shown in this study.

A study by Mathur et al. used CPRD data with a nationally representative sample of people with T2DM in the UK and reported the difference of DR and STDR incidence by ethnicity. According to this study, there was no statistically significant difference in incidence of DR among ethnic minority groups compared to White ethnic groups, however in relation to STDR, South Asians had significantly higher risk compared to White ethnic groups (HR: 1.25 95% CI 1.00–1.56) after adjustment for age, duration of diabetes, gender, deprivation and UK region [4]. Even though the CPRD study had assessed differences in DR and STDR incidence by ethnicity, one of the main limitations of this dataset was poor representation of minor ethnic groups due to lack of recording of ethnicity in CPRD data.

When we consider prevalence studies, higher prevalence of DR among South Asians and Africans have been observed compared to White ethnic groups [25]. A study that aimed to provide estimates of visual impairment (including DR) among people attending DR screening in the UK in 2012, showed that South Asians (OR 1.10, 95% CI 1.02 to 1.18) and Black ethnic groups (OR 1.79, 95CI 1.70 to 1.89) have higher prevalence of visual impairment compared to White groups [25]. Another study from the UK on DR screening has identified younger age, social deprivation, ethnicity and duration of diabetes as independent risk factor of non-attendance, and referable retinopathy confirmed the same association of increase risk of referable DR among Asians (OR 1.57, 95%CI 1.43 to 1.72) and Black (OR 1.65, 95% CI 1.49–1.83) ethnic groups compared to White groups even after adjusting for age, gender, deprivation, diabetes duration and type [26]. All these findings suggest that extra efforts should be made to identify and treat STDR in these ethnic groups and to ensure that recording of self-reported ethnic group is implemented. It is now a contractual obligation in the 2020 general practice contract and is also a requirement of hospital data [27,28]. East London general practitioners have successfully prioritised recording of self-reported ethnic group over three decades [29].

Previous studies have suggested that higher prevalence of DR and STDR among ethnic minorities is likely to be due to suboptimal control of risk factors, delayed attendance at DR screening programmes and poor treatment outcomes compared to White ethnic groups in the UK [30]. However, the role of ethnicity as an independent risk factor in developing DR and STDR is less well-researched mainly due to the lack of data from cohorts representing the three main ethnic groups in the UK. In this study, we’ve adjusted for all known risk factors that are found to be associated with DR and STDR and found that ethnicity is an independent risk factor for incident DR and STDR with higher risk among ethnic minorities. The initial univariable analysis showed that higher proportion of South Asians were younger, affluent and had lower blood pressure compared to other groups. Despite these characteristics of South Asians at study baseline, adjustment for all relevant covariates in the multivariable analysis revealed that they have a higher risk of DR and STDR compared to Whites and other groups.

We also ensured generalisability of our study results by utilising routinely collected data from anonymised electronic datasets of patients in the NHS in the UK. The NHS is also free to all at the point of delivery, reducing inequalities in accessing healthcare. Another strength of our study is that the primary care datasets we used also had more than 98% of completeness in relation to self-reported ethnicity recording. This routinely collected primary care data set is regularly updated and therefore can be used to provide timely information on demographic makeup of the general population in the relevant area. Moreover, the data used in this study was collected prospectively and therefore the data are less likely to be affected by recall bias, or observer bias which could be issues in survey data or retrospectively collected data.

Routinely collected primary care data only provides what is recorded by the clinician and therefore, in this dataset some of the data might be missing if they were not recorded. Data recording practices in general practice settings may also vary based on different financial incentivisation packages. In addition, incomplete data and coding errors can occur. However, we have addressed these issues by checking the data for any implausible values and excluding them and also having missing data as a separate category within the data analysis to keep any bias to a minimum.

## 5. Conclusions

Our study illustrates the higher risk of DR and STDR in people with T2DM from ethnic minority groups. These groups also have a higher prevalence of T2DM, further increasing their risk of DR and STDR compared to the White population. Retinopathy is another adverse health outcome therefore that is more common in people with T2DM from ethnic minority groups. Our findings illustrate the importance of improving the prevention, early diagnosis and management of T2DM in the UK to reduce the burden of ill-health from retinopathy and the other adverse outcomes of T2DM, particularly in people from ethnic minority groups.

## Figures and Tables

**Table 1:** Baseline characteristics of the study population—People with newly diagnosed type 2 diabetes.

	Whole Population	White	South Asian	Black	Other
	N = 32,652	N = 8727	N = 15,291	N = 6639	N = 1995
>Age at T2DM diagnosis, years					
<45	10,136 (31.0)	1423 (16.3)	6536 (42.7)	1678 (25.3)	499 (25.0)
45 to <55	9352 (28.6)	2258 (25.9)	4329 (28.3)	2176 (32.8)	589 (29.5)
55 to <65	6789 (20.8)	2360 (27.0)	2530 (16.6)	1369 (20.6)	530 (26.6)
65 to <75	4091 (12.5)	1622 (18.6)	1269 (8.3)	947 (14.3)	253 (12.7)
75+	2284 (6.9)	1064 (12.2)	627 (4.1)	469 (7.1)	124 (6.2)
Gender					
Male	18,332 (56.1)	5064 (58.0)	8842 (57.8)	3381 (50.9)	1045 (52.4)
Female	14,320 (43.8)	3663 (41.9)	6449 (42.2)	3258 (49.1)	950 (47.7)
Townsend Score (quintiles)					
1 (most affluent)	6874 (21.0)	1484 (17.0)	4043 (26.4)	978 (14.7)	369 (18.5)
2	6795 (20.8)	1766 (20.2)	3159 (20.7)	1425 (21.5)	445 (22.3)
3	6409 (19.6)	1931 (22.1)	2708 (17.7)	1369 (20.6)	401 (20.1)
4	6335 (19.4)	1774 (20.3)	2807 (18.4)	1390 (20.9)	364 (18.3)
5 (most deprived)	6130 (18.8)	1739 (19.9)	2535 (16.6)	1448 (21.8)	408 (20.4)
Not recorded	109 (0.3)	33 (0.4)	39 (0.3)	29 (0.4)	8 (0.4)
Body Mass Index (kg/m^2^)					
<18.5	321(1.0)	71 (0.8)	167 (1.1)	51 (0.8)	32 (1.6)
18.5 to <25	4627 (14.2)	686 (7.9)	3002 (19.6)	610 (9.2)	329 (16.5)
25 to <30	10,979 (33.7)	1990 (22.8)	6381 (41.7)	1951 (29.4)	657 (32.9)
≥30	14067 (43.1)	5094 (58.4)	4706 (30.8)	3494 (52.6)	773 (38.7)
Not recorded	2658 (8.2)	886 (10.2)	1035 (6.8)	533 (8.0)	204 (10.3)
HbA1c (mmol/mol)					
<50	6927 (21.2)	2198 (25.2)	2912 (19.0)	1399 (21.1)	418 (20.9)
50 to <100	18, 368 (56.2)	4626 (53.0)	9002 (58.9)	3602 (54.3)	1138 (57.0)
≥100	2561 (7.8)	627 (7.6)	970 (6.3)	795 (11.9)	169 (8.5)
Not recorded	4796 (14.7)	1276 (14.6)	2407 (15.7)	843 (12.7)	270 (13.5)
Systolic Blood Pressure (mmHg)					
<120	6396 (19.6)	1224 (14.0)	3897 (25.5)	918 (13.8)	357 (17.9)
120 to <130	7537 (23.1)	1891 (21.7)	3865 (25.3)	1315 (20.2)	430 (21.6)
130 to <140	8468 (25.9)	2404 (27.5)	3756 (24.6)	1792 (27.0)	516 (25.8)
≥140	9273 (28.4)	2943 (33.7)	3348 (22.0)	2367 (35.6)	615 (30.8)
Not recorded	978 (3.0)	265 (3.0)	425 (2.8)	211 (3.2)	77 (3.9)
Total Cholesterol (mmol/L)					
<5.2	17,072 (52.3)	4537 (52.0)	8221 (53.8)	3351 (50.5)	963 (48.3)
5.2 to <6.2	8188 (25.1)	2068 (23.7)	3898 (25.5)	1719 (25.9)	503 (25.2)
≥6.2	5413 (16.6)	1542 (17.7)	2359 (15.4)	1127 (16.8)	385 (19.3)
Not recorded	1979 (6.1)	580 (6.6)	813 (5.3)	442 (6.7)	144 (7.2)
eGFR (mL/min/1.73m^2^)					
<60	2546 (7.8)	939 (10.8)	738 (4.8)	747 (11.3)	122 (6.2)
≥60	27,832 (85.2)	7219 (82.7)	13,461 (88.0)	5438 (81.9)	1714 (85.9)
Not recorded	2274 (7.1)	569 (6.5)	1092 (7.1)	454 (6.8)	159 (7.9)
History of Cardiovascular Disease					
No	29,532 (90.4)	7339 (84.1)	14,135 (92.4)	6233 (93.8)	1825 (91.5)
Yes	3120 (9.6)	1388 (15.9)	1156 (7.4)	406 (6.2)	170 (8.5)
History of Antidiabetic	21,779 (66.7)	6002 (66.8)	10,037 (65.6)	4409 (66.4)	1331 (66.7)
No drug	10,873 (31.3)	2725 (31.2)	5254 (34.4)	2230 (33.6)	664 (33.3)
One drug					
History of Antihypertensive Medication					
No	17,620 (53.9)	3935 (45.1)	9396 (61.4)	3232 (48.7)	1057 (53.0)
Yes	15,032 (46.0)	4792 (54.9)	5895 (38.6)	3407 (51.3)	938 (47.0)
History of Statin Medication					
No	6229 (19.1)	1495 (17.1)	2553 (16.7)	1749 (26.3)	432 (21.7)
Yes	26,423 (80.9)	7232 (82.9)	12,738 (83.5)	4890 (73.7)	1563 (78.3)

Abbreviations: eGFR-Estimated glomerular filtration rate. ‘Other’ group: this group included Chinese, other Asian, other Black and people belong to any other ethnicity.

**Table 2:** Baseline characteristics of the study population—people with known type 2 diabetes.

	Whole Population	White	South Asian	Black	Other
	N = 25,564	N = 6946	N = 11,963	N = 5216	N = 1439
Age, Years					
<45	4956 (19.4)	738 (10.6)	3055 (25.5)	923 (17.7)	243 (16.9)
45 to <55	6207 (24.3)	1357 (19.5)	3297 (27.5)	1213 (23.3)	340 (23.7)
55 to <65	5860 (22.9)	1827 (26.3)	2495 (20.8)	1134 (21.7)	404 (28.0)
65 to <75	5407 (21.1)	1628 (23.4)	2218 (18.5)	1258 (24.1)	303 (21.0)
75+	3134 (12.2)	1396 (20.1)	901 (7.5)	688 (13.2)	149 (10.3)
Duration of Diabetes					
0 to <2 years (ref)	4030 (15.8)	1089 (15.7)	1875 (15.7)	806 (15.5)	260 (18.1)
2 to <5 years	6835 (26.7)	1891 (27.2)	3177 (26.6)	1368 (26.2)	399 (27.7)
5 to <10 years	8125 (31.8)	2281 (32.8)	3799 (31.7)	1642 (31.5)	403 (28.0)
≥10 years	6574 (25.7)	1685 (24.3)	3112 (26.0)	1400 (26.8)	377 (26.1)
Gender					
Male	13,289 (51.9)	3724 (53.6)	6333 (52.9)	2524 (48.4)	708 (49.2)
Female	12,275 (48.0)	3222 (46.4)	5630 (47.1)	2692 (51.6)	731 (50.8)
Townsend Score (quintiles)					
1	4812 (18.8)	1016 (14.6)	2774 (23.2)	762 (14.6)	260 (18.0)
2	4840 (18.9)	1270 (18.3)	2264 (18.9)	1021 (19.6)	285 (19.8)
3	5110 (20.0)	1622 (23.4)	2161 (18.3)	1036 (19.9)	291 (20.2)
4	5366 (20.9)	1537 (22.1)	2432 (20.3)	1151 (22.1)	246 (17.1)
5	5373 (21.0)	1488 (21.4)	2307 (19.3)	1227 (23.5)	351 (24.4)
Not recorded	63 (0.3)	13 (0.2)	25 (0.2)	19 (0.4)	6 (0.4)
Body Mass Index (kg/m^2^)					
<18.5	389 (1.5)	83 (1.2)	216 (1.8)	56 (1.1)	34 (2.4)
18.5 to <25	4608 (18.0)	646 (9.3)	2999 (25.1)	675 (12.9)	288 (20.0)
25 to <30	8745 (34.2)	1778 (25.6)	4788 (40.0)	1723 (33.0)	456 (31.6)
≥30	8966 (35.1)	3466 (49.9)	2814 (23.5)	2191 (42.0)	495 (34.4)
Not recorded	2856 (11.2)	973 (14.0)	1146 (9.6)	571 (10.9)	166 (11.5)
HbA1c (mmol/mol)					
<50	5692 (22.3)	1897 (27.3)	2307 (19.3)	1177 (22.6)	312 (21.7)
50 to <100	15,362 (60.1)	3794 (54.6)	7701 (64.4)	3005 (57.6)	862 (59.9)
≥100	1644 (6.4)	373 (5.4)	702 (5.8)	474 (9.1)	95 (6.6)
Not recorded	2866 (11.2)	882 (12.7)	1254 (10.5)	560 (10.7)	170 (11.8)
Systolic Blood Pressure (mmHg)					
<120	5347 (20.9)	1129 (16.2)	3166 (26.5)	774 (14.8)	279 (19.4)
120 to <130	5733 (22.4)	1436 (20.7)	2945 (24.6)	1058 (20.3)	294 (20.5)
130 to <140	6313 (24.7)	1887 (27.2)	2699 (22.5)	1365 (26.2)	362 (25.2)
≥140	7600 (29.7)	2318 (33.3)	2929 (24.5)	1881 (36.0)	472 (32.7)
Not recorded	571 (2.2)	176 (2.5)	225 (1.9)	138 (2.7)	32 (2.2)
Total Cholesterol (mmol/L)					
<5.2	18,456 (72.2)	4904 (70.6)	8949 (74.8)	3605 (69.1)	998 (69.4)
5.2 to <6.2	3237 (12.7)	883 (12.7)	1388 (11.6)	767 (14.7)	199 (13.8)
≥6.2	1643 (6.4)	465 (6.7)	677 (5.7)	376 (7.2)	125 (8.7)
Not recorded	2228 (8.7)	694 (9.9)	949 (7.9)	468 (8.9)	117 (8.1)
eGFR (mL/min/1.73m^2^)					
<60	2978 (11.7)	1036 (14.9)	1126 (9.4)	678 (13.0)	138 (9.6)
≥60	20,160 (78.8)	5140 (74.0)	9813 (82.0)	4039 (77.4)	1168 (81.2)
Not recorded	2426 (9.5)	770 (11.1)	1024 (8.6)	499 (9.6)	133 (9.2)
History of Cardiovascular Disease					
No	21,734 (85.0)	5474 (78.8)	10,338 (86.4)	4669 (89.5)	1253 (87.1)
Yes	3830 (15.0)	1472 (21.2)	1625 (13.6)	547 (10.5)	186 (12.9)
History of Antidiabetic Drugs					
No drug	4470 (17.5)	1508 (21.7)	1750 (14.6)	954 (18.3)	258 (17.9)
One drug	7244 (28.3)	1988 (28.6)	3414 (28.5)	1403 (26.9)	439 (30.5)
Two drugs	9356 (36.6)	2242 (32.3)	4781 (40.0)	1832 (35.1)	501 (34.8)
Insulin	4494 (17.6)	1208 (17.4)	2018 (16.9)	1027 (19.7)	241 (16.8)
History of Antihypertensive Medication					
No	8880 (34.7)	2003 (28.8)	4562 (38.1)	1770 (33.9)	545 (37.9)
Yes	16,684 (65.3)	4943 (71.2)	7401 (61.9)	3446 (66.1)	894 (62.1)
History of Statin Medication—ever					
No	2528 (9.9)	741 (10.7)	763 (6.4)	831 (15.9)	193 (13.4)
Yes	23,036 (90.1)	6205 (89.3)	11,200 (93.6)	4385 (84.1)	1246 (86.6)

Abbreviations: eGFR-Estimated glomerular filtration rate. ‘Other’ group: this group included Chinese, other Asian, other Black and people belong to any other ethnicity.

**Table 3:** Number of incident cases of DR/STDR during 10-year follow-up and incidence rates per 1000 person years in newly diagnosed and known T2DM at baseline.

People with Newly Diagnosed T2DM at Baseline									
Ethnicity	Total Number of People	DR				STDR			
		Number of Events	Percentage with Events	Person-Years	Incidence Rate per 1000 Person Years (95% CI)	Number of Events	Percentage with Events	Person-Years	Incidence Rate per 1000 Person Years (95% CI)
Whole Population	32,652	8638	26.5	104,257.8	82.85 (81.12–84.61)	557	1.7	132,126.7	4.21 (3.88–4.58)
White	8727	2227	25.5	29,624.4	75.17 (72.12–78.36)	137	1.6	37,185.5	3.68 (3.11–4.35)
South Asian	15,291	4035	26.4	47,448.6	85.04 (82.45–87.70)	229	1.5	60,163.5	3.81 (3.34–4.33)
Black	6639	1833	27.6	20,953.3	87.48 (83.56–91.58)	152	2.3	26,854.9	5.66 (4.83–6.63)
Other	1995	543	27.2	6231.5	87.13 (80.11–94.78)	39	1.9	7922.8	4.92 (3.59–6.73)
**People with Known T2DM at Baseline**									
**Ethnicity**	**Total Number of People**	**DR**				**STDR**			
		**Number of Events**	**Percentage with Events**	**Person Years**	**Incidence Rate per 1000 Person Years (95% CI)**	**Number of Events**	**Percentage with Events**	**Person Years**	**Incidence Rate per 1000 Person Years (95% CI)**
Whole Population	25,564	12,124	47.4	89,534.1	135.41 (133.02–137.84)	2200	8.6	137,140.3	16.04 (15.38–16. 72)
White	6946	2946	42.4	25,900.1	113.74 (109.71–117.92)	454	6.5	37,727.6	12.03 (10.97–13.19)
South Asian	11,963	6100	50.9	40,447.5	150.81 (147.07–154.64)	1145	9.6	64,136.8	17.85 (16.84–18.91)
Black	5216	2422	46.4	18,134.7	133.56 (128.37–138.98)	480	9.2	27,699.3	17.32 (15.84–18.94)
Other	1439	656	45.6	5051.9	129.85 (120.28–140.18)	121	8.4	7576.5	15.97 (13.36–19.08)

**Table 4:** Multivariable Cox regression analysis of outcome measures in newly diagnosed diabetes—Newly diagnosed T2DM Cases.

	DR		STDR	
	Adjusted HR	*p* Value	Adjusted HR	*p* Value
Ethnic Group				
White (ref)	1		1	
South Asian				
Indian	1.10 (1.01–1.19)	0.029	0.86 (0.60–1.21)	0.377
Pakistani	1.14 (1.03–1.25)	0.011	1.36 (0.95–1.94)	0.094
Bangladeshi	1.02 (0.95–1.09)	0.733	0.80 (0.61–1.05)	0.107
Black				
Caribbean	1.07 (0.98–1.17)	0.178	1.18 (0.85–1.65)	0.315
African	1.16 (1.07–1.26)	<0.001	1.36 (1.02–1.83)	0.039
Mixed and other				
Mixed	1.02 (0.86–1.21)	0.878	1.37 (0.76–2.48)	0.296
Other	1.10 (1.03–1.19)	0.012	1.26 (0.95–1.67)	0.108
Age at Study Entry, Years				
<45	1.21 (1.09–1.35)	0.001	0.84 (0.56–1.25)	0.392
45–54	1.15 (1.04–1.27)	0.011	0.98 (0.67–1.43)	0.897
55–64	1.10 (0.99–1.22)	0.089	0.79 (0.54–1.17)	0.236
65–74	0.99 (0.89–1.10)	0.728	0.93 (0.63–1.38)	0.735
75+	1		1	
Gender				
Female	1		1	
Male	1.12(1.07–1.17)	<0.001	1.22 (1.02–1.47)	0.001
Townsend Score (quintiles)				
1 (affluent)	1		1	
2	0.93 (0.88–1.00)	0.028	0.83 (0.63–1.09)	0.176
3	0.95 (0.88–1.01)	0.072	1.05 (0.80–1.38)	0.724
4	0.95 (0.89–1.02)	0.105	1.24 (0.96–1.62)	0.104
5 (deprived)	0.90 (0.84–0.97)	0.002	1.18 (0.90–1.54)	0.229
Not recorded	0.58 (0.38–0.90)	0.015	0.51 (0.07–3.63)	0.497
Body Mass Index (kg/m^2^)				
<18.5	1		1	
18.5–25	1.00 (0.81–1.24)	0.97	0.66 (0.37–1.17)	0.157
25–30	0.90 (0.73–1.12)	0.322	0.47 (0.27–0.82)	0.008
≥30	0.87 (0.70–1.07)	0.172	0.32 (0.18–0.57)	<0.001
Not recorded	0.73 (0.58–0.91)	0.005	0.24 (0.12–0.48)	<0.001
HbA1c (mmol/mol)				
<50	1		1	
50–99	1.19 (1.12–1.26)	<0.001	1.43 (1.10–1.84)	0.007
≥ 100	1.70 (1.57–1.85)	<0.001	3.68 (2.73–4.95)	<0.001
Not recorded	1.07 (0.98–1.16)	0.143	1.31 (0.92–1.87)	0.13
Systolic Blood Pressure—SBP (mmHg)				
<120	1		1	
120–129	1.05 (0.98–1.12)	0.237	1.13 (0.85–1.51)	0.385
130–140	1.07 (1.01–1.15)	0.05	1.23 (0.93–1.62)	0.147
≥140	1.25 (1.17–1.33)	<0.001	1.88 (1.45–2.44)	<0.001
Not recorded	0.95 (0.80–1.14)	0.551	0.98 (0.47–2.02)	0.947
Total Cholesterol (mmol/L)				
<5.2	1		1	
5.2- 6.1	0.97 (0.92–1.02)	0.195	0.83 (0.67–1.02)	0.081
≥6.2	1.01 (0.95–1.08)	0.795	0.94 (0.75–1.19)	0.622
Not recorded	0.91 (0.80–1.03)	0.13	1.37 (0.89–2.11)	0.149
eGFR (mL/min/1.73m^2^)				
≥60	1		1	
<60	1.09 (1.00–1.19)	0.057	1.32 (0.97–1.79)	0.077
Not recorded	1.00 (0.90–1.12)	0.968	1.20 (0.80–1.79)	0.375
Cardiovascular Disease History—ever				
No	1		1	
Yes	1.05 (0.97–1.13)	0.304	1.06 (0.77–1.46)	0.722
Antidiabetic Drugs History—ever Closest Record to Baseline				
No drug	1		1	
One drug	0.97 (0.93–1.02)	0.142	0.97 (0.81–1.16)	0.725
History of Antihypertensive Medication				
No	1		1	
Yes	1.02 (0.97–1.07)	0.495	0.77 (0.63–0.93)	0.008
Statin History				
No	1		1	
Yes	1.12 (1.07–1.18)	<0.001	1.20 (0.99–1.46)	0.066

Abbreviations: eGFR-Estimated glomerular filtration rate. ‘Other’ group: this group included Chinese, other Asian, other Black and people belong to any other ethnicity.

**Table 5:** Multivariable Cox regression analysis of outcome measures in people with known diabetes—KnownT2DM Cases.

	DR		STDR	
	Adjusted HR	*p* Value	Adjusted HR	*p* Value
Ethnic Group				
White (ref)	1		1	
South Asian				
Indian	1.24 (1.16–1.33)	<0.001	1.39 (1.18–1.63)	<0.001
Pakistani	1.20 (1.11–1.30)	<0.001	1.28 (1.05–1.55)	0.016
Bangladeshi	1.11 (1.05–1.17)	<0.001	1.36 (1.19–1.55)	<0.001
Black				
Caribbean	1.09 (1.01–1.16)	0.02	1.22 (1.04–1.43)	0.016
African	1.06 (0.98–1.14)	0.145	1.10 (0.92–1.33)	0.294
Mixed and Other				
Mixed	1.10 (0.96–1.26)	0.19	1.17 (0.85–1.61)	0.328
Other	1.03 (0.97–1.11)	0.335	1.25 (1.07–1.47)	0.006
Duration of Diabetes				
0 to <2 years (ref)	1		1	
2 to <5 years	1.17 (1.10–1.24)	<0.001	1.18 (0.99–1.40)	0.059
5 to <10 years	1.37 (1.29–1.45)	<0.001	1.40 (1.18–1.66)	<0.001
≥10 years	1.83 (1.71–1.96)	<0.001	2.46 (2.06–2.90)	<0.001
Age at Study Entry, Years				
<45	1.52 (1.40–1.66)	<0.001	1.68 (1.37–2.05)	<0.001
45–54	1.51 (1.40–1.62)	<0.001	1.91 (1.60–2.29)	<0.001
55–64	1.32 (1.23–1.42)	<0.001	1.41 (1.18–1.68)	<0.001
65–74	1.15 (1.07–1.23)	<0.001	1.35 (1.14–1.61)	0.001
75+	1		1	
Gender				
Female	1		1	
Male	1.10 (1.06–1.14)	<0.001	1.15 (1.06–1.26)	0.001
Townsend Score (quintiles)				
1	1		1	
2	0.95 (0.89–1.00)	0.067	1.06 (0.91–1.22)	0.495
3	0.97 (0.91–1.02)	0.247	1.22 (1.06–1.40)	0.005
4	0.91 (0.86–0.97)	0.003	1.22 (1.06–1.40)	0.005
5	0.95 (0.89–1.00)	0.088	1.30 (1.13–1.49)	<0.001
Not recorded	0.86 (0.58–1.28)	0.462	0.46 (0.11–1.88)	0.286
Body Mass Index (kg/m^2^)				
<18.5	1		1	
18.5–25	1.03 (0.88–1.20)	0.703	0.94 (0.66–1.35)	0.756
25–30	1.00 (0.86–1.18)	0.94	0.89 (0.63–1.27)	0.551
≥30	0.91 (0.78–1.07)	0.261	0.76 (0.53–1.08)	0.132
Not recorded	0.96 (0.81–1.13)	0.63	1.06 (0.74–1.53)	0.72
HbA1c (mmol/mol)				
<50	1		1	
50–99	1.29 (1.23–1.36)	<0.001	1.62 (1.40–1.87)	<0.001
≥100	1.83 (1.69–1.98)	<0.001	3.20 (2.68–3.83)	<0.001
Not recorded	1.00 (0.92–1.09)	0.905	1.53 (1.25–1.88)	<0.001
Systolic Blood Pressure—SBP (mmHg)				
<120	1		1	
120–129	1.12 (1.05–1.18)	<0.001	1.26 (1.09–1.45)	0.001
130–140	1.16 (1.10–1.23)	<0.001	1.34 (1.17–1.55)	<0.001
≥140	1.28 (1.21–1.35)	<0.001	1.80 (1.57–2.05)	<0.001
Not recorded	0.97 (0.84–1.13)	0.709	1.17 (0.83–1.63)	0.356
Total Cholesterol (mmol/L)				
<5.2	1		1	
5.2–6.1	0.96 (0.91–1.02)	0.197	1.01 (0.88–1.15)	0.871
≥6.2	0.96 (0.89–1.03)	0.233	1.04 (0.88–1.24)	0.593
Not recorded	0.99 (0.91–1.07)	0.857	1.07 (0.89–1.27)	0.473
eGFR (mL/min/1.73m^2^)				
≥60	1		1	
<60	1.10 (1.04–1.17)	0.001	1.35 (1.19–1.54)	<0.001
Not recorded	1.05 (0.98–1.14)	0.178	1.10 (0.93–1.30)	0.279
History of Cardiovascular Disease				
No	1		1	
Yes	0.98 (0.93–1.04)	0.584	0.96 (0.85–1.08)	0.514
History of Antidiabetic Drugs History-				
No drug	1		1	
One drug	1.01 (0.94–1.07)	0.854	0.82 (0.68–0.98)	0.035
Two drugs	1.26 (1.18–1.34)	<0.001	1.36 (1.15–1.60)	<0.001
Insulin	1.77 (1.65–1.89)	<0.001	2.72 (2.30–3.21)	<0.001
History of Antihypertensives				
No	1		1	
Yes	0.99 (0.95–1.04)	0.752	1.00 (0.89–1.12)	0.993
History of Statin				
No	1		1	
Yes	1.07 (1.01–1.13)	0.008	0.96 (0.85–1.08)	0.519

Abbreviations: eGFR- Estimated glomerular filtration rate. ‘Other’ group: this group included Chinese, other Asian, other Black and people belong to any other ethnicity.

## Data Availability

Restrictions apply to the availability of this data. This study was conducted using de-identified data from general practice electronic health records collected in three Clinical Commissioning Groups (CCGs) in East London and data are available with permission from the Caldicott guardian of this anonymised dataset in Queen Mary University London (QMUL).
